# Entropy in specificity and thermodynamics of binding

**DOI:** 10.1186/1758-2946-6-S1-O8

**Published:** 2014-03-11

**Authors:** Klaus R Liedl

**Affiliations:** 1 Leopold-Franzens-University Innsbruck, Faculty of Chemistry and Pharmacy, Centre for Chemistry and Biomedicine (CCB), Innrain 82, 6020 Innsbruck, Austria

## 

Entropy is an elusive and somehow non-intuitive concept. Nevertheless, entropy governs spontaneous thermodynamic processes as important contribution to Gibbs Free Energy. Information theory defines Shannon entropy as a measure for uncertainty. In the context of protein binding the inherent link between flexibility, thus conformational entropy, and substrate specificity is discussed. Substrate promiscuity of proteases is quantified as cleavage entropy correlating local binding site flexibility directly with substrate readout. Caspases are examined as example protease family, where active site dynamics play a major role in mediating substrate specificity. Direct comparison of entropy in substrate data allows highlighting previously unexpected similarities in substrate recognition in proteases. Promiscuous binding to several protease targets demonstrates the emerging importance of quantitative studies on binding specificity. Shannon entropy applied to probability densities is used to rationalize ordering or disordering by binding processes. We have developed a data-driven method to reconstruct probability densities from discrete sampling by computer simulations. Application to solvent degrees of freedom leads to excellent correlation with experimental data.

